# Integrated multi-omics analysis reveals diagnostic biomarkers and therapeutic targets for systemic lupus erythematosus

**DOI:** 10.1097/MD.0000000000042290

**Published:** 2025-08-15

**Authors:** Luofei Huang, Jian Shi, Han Li, Quanzhi Lin

**Affiliations:** a Liuzhou Municipal Liutie Central Hospital, Liuzhou, China; b Department of Internal Medicine, The People’s Hospital of Laibin, Laibin, Guangxi, China; c Department of Internal Medicine, Liuzhou People’s Hospital, Liuzhou, Guangxi, China; d Department of Internal Medicine, The First Affiliated Hospital of Guangxi University of Science and Technology, Liuzhou, Guangxi, China.

**Keywords:** immune infiltration, Mendelian randomization, molecular docking, plasma proteins, systemic lupus erythematosus

## Abstract

Systemic lupus erythematosus (SLE) is a complex autoimmune disorder with poorly understood molecular mechanisms. This study investigated causal relationships between plasma proteins and SLE using two-sample Mendelian randomization analysis based on genome-wide association study data. Functional enrichment analyses Gene Ontology and Kyoto Encyclopedia of Genes and Genomes and protein–protein interaction network analysis identified apolipoprotein A2, MANSC domain-containing protein 1, and proteasome subunit beta 5 as key genes involved in glycoprotein metabolism and immune responses. Molecular docking simulations suggested meglumine and vorinostat as potential therapeutic candidates. A diagnostic nomogram model incorporating these genes demonstrated high predictive accuracy (area under the curve = 0.968). Single-cell RNA sequencing revealed that proteasome subunit beta 5 and MANSC domain-containing protein 1 were predominantly expressed in T cells and monocytes, implicating their roles in SLE-associated immune dysregulation. These findings highlight novel biomarkers, diagnostic tools, and therapeutic targets for SLE, advancing our understanding of its pathogenesis and treatment.

## 1. Introduction

Systemic lupus erythematosus (SLE) is a complex, multisystem autoimmune disorder characterized by loss of immune tolerance, persistent inflammation, and heterogeneous clinical manifestations. The disease pathogenesis involves aberrant B-cell and T-cell activation, autoantibody production, and immune complex deposition, culminating in progressive damage to multiple organ systems including the kidneys (lupus nephritis), cardiovascular system, skin, and hematological components. This systemic involvement, coupled with the disease’s fluctuating course between remission and flares, presents significant diagnostic and therapeutic challenges in clinical management.^[1,2]^ The disease primarily affects women of childbearing age and is characterized by periods of exacerbation and remission.^[3]^ Despite advancements in understanding SLE’s pathogenesis, the exact mechanisms remain unclear. Epidemiological studies have shown that SLE prevalence varies across different populations, with higher rates observed in individuals of African, Hispanic, and Asian descent.^[4]^ Current treatment strategies focus on immunosuppressive agents, including corticosteroids, antimalarial drugs, and biologics, aimed at controlling inflammation and preventing organ damage.^[5,6]^ However, challenges such as drug resistance, side effects, and the heterogeneous nature of the disease hinder effective long-term management and early diagnosis.^[7]^

Recent research has highlighted the potential role of plasma proteins as key players in the pathogenesis of SLE.^[8]^ These proteins, influenced by genetic variants, serve as biomarkers for disease risk, progression, and response to treatment.^[9]^ Plasma protein levels are intricately linked to immune system modulation, tissue inflammation, and autoantibody production, all of which are hallmarks of SLE.^[10]^ However, the exact mechanisms by which plasma proteins interact with the immune system in SLE remain poorly understood. Mendelian randomization (MR) analysis has emerged as a powerful tool to investigate causal relationships between genetic variants associated with plasma proteins and SLE susceptibility In many previous studies,^[11,12]^ offering insights into how these proteins may contribute to disease onset and progression.^[13,14]^ The identification of specific plasma proteins involved in SLE may also open new avenues for therapeutic interventions, where modulating the levels or activity of these proteins could potentially mitigate disease severity. Thus, understanding the complex interaction between plasma proteins and the immune system is crucial for developing targeted and personalized treatments for SLE.

This study investigates the genetic associations between plasma proteins and SLE risk using analysis and functional enrichment studies. We further employ protein–protein interaction (PPI) networks and single-cell RNA sequencing (scRNA-seq) to identify key signature genes implicated in SLE pathogenesis. By integrating these advanced approaches, we develop a diagnostic model to accurately predict SLE risk based on genetic markers. Ultimately, our findings aim to advance personalized SLE therapeutics by uncovering novel biomarkers and therapeutic targets. This work underscores the dual potential of plasma proteins (as diagnostic tools and therapeutic targets) while providing mechanistic insights into SLE’s genetic and immune dysregulation.

## 2. Materials and methods

### 2.1. Source of data

The plasma protein data primarily come from a genome-wide association study (GWAS) analysis of plasma protein levels in 35,559 Icelandic individuals, identifying 18,084 protein quantitative trait loci associated with genetic variants, of which 19% were rare variants. This study also revealed 257,490 associations between plasma protein levels and 373 diseases and traits.^[15]^ For the SLE data, we obtained genetic association information from the IEU Open GWAS project (https://gwas.mrcieu.ac.uk/). The dataset for SLE included 647 cases and 482,264 controls, all of European ancestry.^[16]^

### 2.2. MR analysis

We conducted a two-sample MR analysis to preliminarily identify plasma proteins potentially linked to the pathogenesis of SLE. MR relies on 3 key assumptions: first, there must be a strong correlation between the instrumental variables and the exposure^[17]^; second, the instrumental variables should be independent of any known or unknown confounders^[18]^; and third, the effects of the instrumental variables on the outcome should be mediated solely through the exposure, not directly.^[19]^ In this study, plasma protein levels were used as the exposure, and SLE as the outcome. To ensure the validity of the instrumental variables, we set the threshold for *P*-values at < 5 × 10⁻⁸, ensuring robust associations with the exposure. To minimize confounding and ensure independence, we applied linkage disequilibrium clustering with an *r*² threshold of < 0.001 to eliminate correlated single nucleotide polymorphisms.^[20]^

### 2.3. Functional enrichment analysis

We performed Gene Ontology (GO) functional annotation and Kyoto Encyclopedia of Genes and Genomes (KEGG) pathway enrichment analysis on the genes associated with the risk of SLE using the enrichment GO and KEGG packages in R^[21,22]^ as previous studies.^[23–25]^ A significance threshold of *P* < .05 was applied to determine statistical relevance. The enriched GO terms were categorized into 3 main ontologies: biological process, cellular component, and molecular function, which provided insights into the biological roles of the SLE risk-related genes. Furthermore, KEGG pathway analysis offered valuable perspectives on the potential cellular pathways involved in the SLE risk-related genes, facilitating a deeper understanding of the underlying molecular mechanisms.

### 2.4. Protein interaction network construction

In this study, we constructed a PPI network using the STRING database and performed analysis and visualization with Cytoscape software^[26]^ as previous studies.^[27]^ Initially, we set the minimum interaction confidence score in STRING to 0.15 while keeping all other parameters at their default values to filter for high-confidence interactions. Next, we conducted degree centrality analysis to identify the top 20 key proteins with the highest degree in the network, which play a central role in the PPI network. Finally, we visualized the network using Cytoscape.

### 2.5. Candidate drug prediction

This study will conduct relevant analyses using the Drug Signatures Database (DSigDB, http://dsigdb.tanlab.org/DSigDBv1.0/). DSigDB is a comprehensive resource that links drugs and compounds to their associated hub genes^[28]^ as previous studies.^[29–31]^ The identified hub genes will be submitted to the database, which will then facilitate the identification of potential drug candidates and allow for the assessment of the pharmacological effects on these target genes.

### 2.6. Molecular docking

In this study, molecular docking simulations were conducted to investigate the interactions between potential drug candidates and target proteins as previous studies.^[32,33]^ The chemical structures of the drug candidates were obtained from the PubChem database (https://pubchem.ncbi.nlm.nih.gov/), which offers comprehensive data on molecular properties, 3D structures, and physicochemical characteristics.^[34]^ The 3D structures of the target proteins were sourced from the RCSB Protein Data Bank (https://www.rcsb.org/), ensuring the use of high-quality, experimentally validated protein models.^[35]^ Docking simulations were carried out using the CB-Dock2 platform (https://cadd.labshare.cn/cb-dock2/index.php), an online tool for protein–ligand docking that automatically identifies binding sites and predicts optimal docking poses.^[36]^ The docking results, including binding affinity and interaction analysis, were then evaluated to assess the stability and potential therapeutic efficacy of the drug candidates.

### 2.7. Selection of signature genes

We will conduct an in-depth analysis of disease-associated genes to identify the trait genes most strongly linked to SLE. To achieve this, we will apply several machine learning techniques, including Random Forest,^[37]^ Support Vector Machine Recursive Feature Elimination (SVM-RFE),^[38]^ and Least Absolute Shrinkage and Selection Operator regression to build a linear model for identifying significant variables^[39]^ as previous studies.^[40,41]^ These methods are widely used for selecting key factors. Finally, we will use a Venn diagram to cross-analyze the results from these methods, allowing us to pinpoint the relevant trait genes associated with SLE.

### 2.8. Construction and validation of the diagnostic model

Based on the identified trait genes, we developed a diagnostic model to predict the risk of developing SLE. The prediction accuracy of the model was assessed using dynamic nomograms, calibration curves, decision curve analysis, and clinical impact curves. Finally, receiver operating characteristic (ROC) curves were generated using R software to further validate the model’s predictive performance.

### 2.9. Gene set variation analysis (GSVA) and gene set enrichment analysis (GSEA)

We employed a non-parametric, unsupervised GSVA approach to identify differentially enriched KEGG pathways associated with SLE.^[42]^ Subsequently, single-gene GSEA was performed for each characteristic gene using the R package clusterProfiler. This allowed us to assess the variations in KEGG pathway enrichment across different gene expression groups.

### 2.10. Immune infiltration analysis was performed using the ssGSEA method

The single-sample gene set enrichment analysis (ssGSEA) technique was utilized to evaluate the extent of immune cell infiltration across the samples.^[43]^ To explore the associations between immune cell populations, immune functions, and signature genes, the “corrplot” package was employed to compute Spearman correlation coefficients and to visually present the findings.

### 2.11. Visualizing the expression of signature genes in single cells using Seurat

To study the expression of signature genes at the single-cell level, we analyzed the publicly available scRNA-seq dataset GSE135779, which includes transcriptomic samples from single PBMCs of both SLE patients and healthy controls. The data were processed and analyzed using the Seurat package in R. Preprocessing steps involved quality control, normalization, and scaling to account for batch effects and other technical variations. Dimensionality reduction was performed using Uniform Manifold Approximation and Projection to visualize cell clusters and their associated gene expression profiles. Subsequently, the expression levels of signature genes were mapped onto the Uniform Manifold Approximation and Projection plot to identify specific cell types or subpopulations that show significant expression of these genes.^[44]^

## 3. Results

### 3.1. Bidirectional MR analysis of plasma proteins and SLE

**Figure 1.**
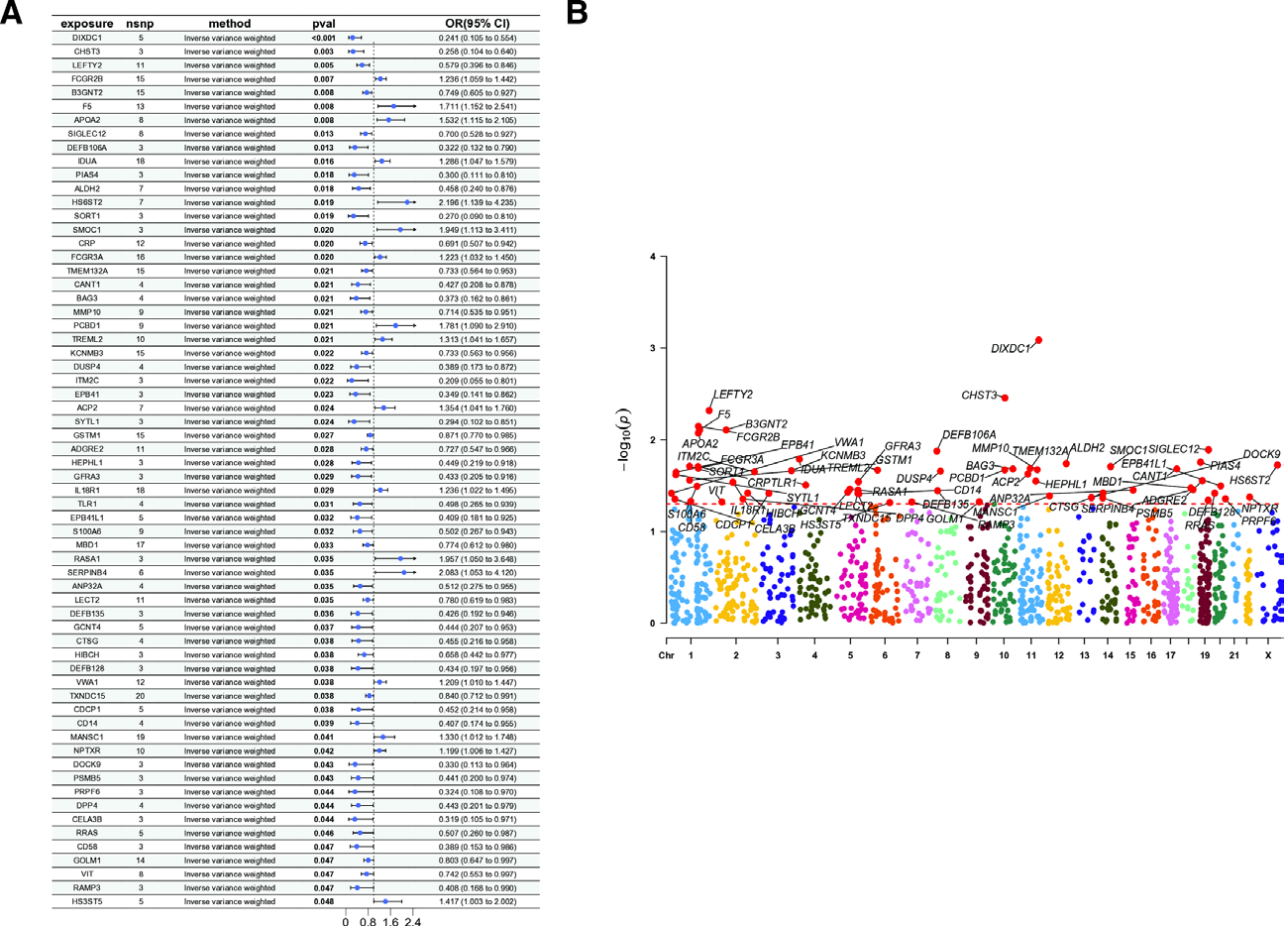
(A) Forest plot showing genes associated with SLE susceptibility; (B) Manhattan plot of genes associated with SLE susceptibility, with key genes highlighted in red. SLE = systemic lupus erythematosus.

We employed two-sample MR analysis with inverse-variance weighted estimation to investigate potential causal relationships between plasma proteins and SLE susceptibility. Our analysis identified several genes significantly associated with SLE risk (Fig. 1A), with additional validation through comprehensive assessments of heterogeneity (Table S1, Supplemental Digital Content, https://links.lww.com/MD/P648) and horizontal pleiotropy confirming the robustness of these causal inferences. The genome-wide distribution of these SLE-associated genes is visually summarized in the Manhattan plot (Fig. 1B), highlighting loci reaching statistical significance across chromosomal regions.

### 3.2. Functional enrichment analysis using GO and KEGG pathway analysis

**Figure 2.**
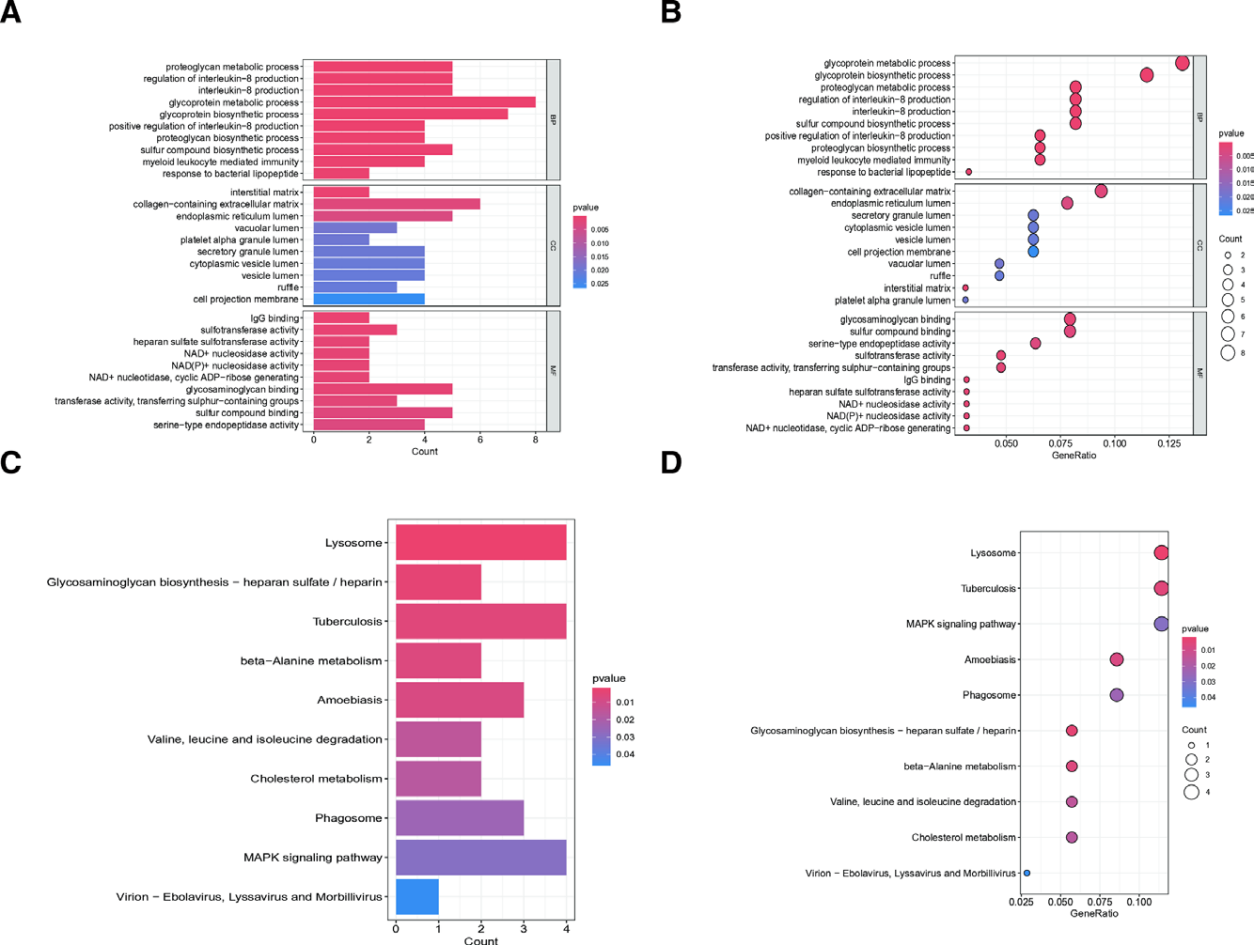
GO and KEGG enrichment analysis of genes associated with SLE susceptibility. (A) Bar plot and (B) bubble plot for GO analysis. (C) Bar plot and (D) bubble plot for KEGG analysis. GO = Gene Ontology, KEGG = Kyoto Encyclopedia of Genes and Genomes, SLE = systemic lupus erythematosus.

To elucidate the biological mechanisms underlying SLE risk genes, we performed comprehensive GO and KEGG enrichment analyses (Fig. 2A–D). The GO analysis revealed 3 major functional categories: (1) biological processes including glycoprotein metabolism/biosynthesis and sulfur compound biosynthesis; (2) cellular components comprising the interstitial matrix and collagen-containing extracellular matrix; and (3) molecular functions such as glycosaminoglycan binding, sulfur compound binding, and sulfotransferase activity (Fig. 2A and B). KEGG pathway analysis identified 4 key pathways significantly associated with SLE risk genes: lysosomal function, heparin sulfate/heparin biosynthesis, tuberculosis infection response, and amebiasis-related pathways (Fig. 2C and D). These findings collectively suggest that SLE pathogenesis involves: (1) extracellular matrix remodeling through collagen and glycosaminoglycan interactions. (2) Sulfur compound metabolic processes that may influence immune regulation. (3) Lysosome-mediated cellular processes potentially linked to autoantigen presentation. (4) Infection–response pathways that could contribute to immune dysregulation. The enrichment of these specific pathways underscores the complex interplay between metabolic processes, immune signaling, and tissue homeostasis in SLE development, providing new insights into potential therapeutic targets.

### 3.3. Selection of HUB genes

**Figure 3.**
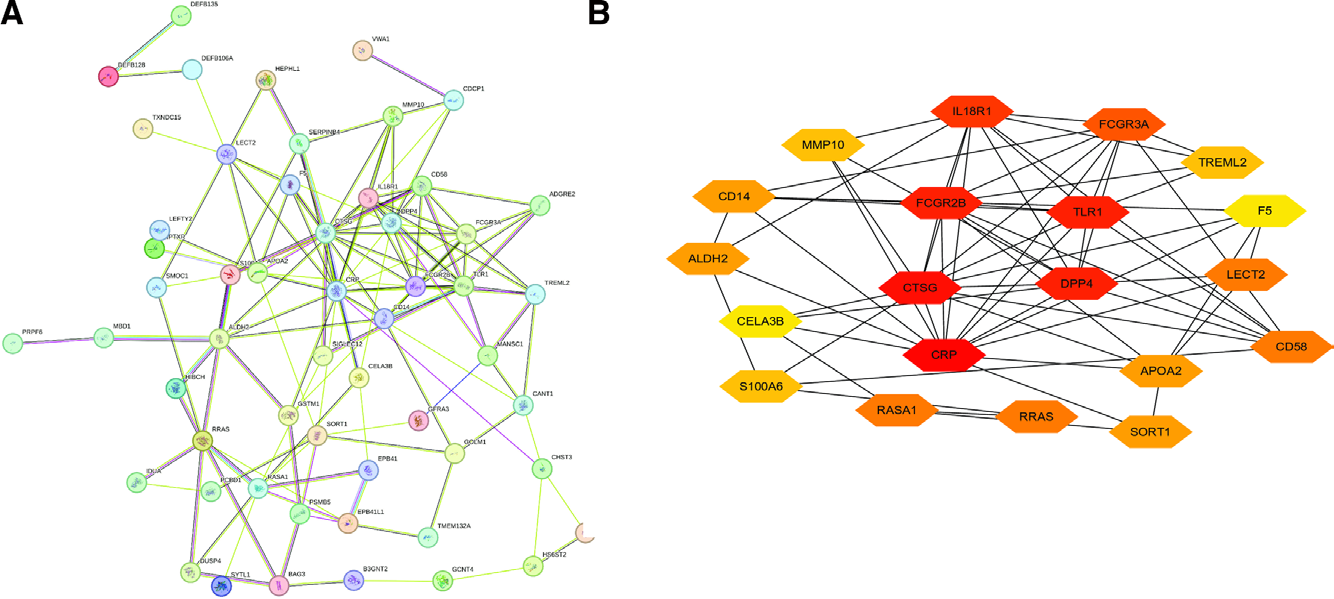
(A) PPI network of proteins associated with SLE susceptibility, constructed using STRING database. (B) Top 20 key proteins with highest degree centrality in the PPI network. PPI = protein–protein interaction, SLE = systemic lupus erythematosus.

We constructed a PPI network using the STRING database, selecting high-confidence protein interactions. After setting the minimum interaction confidence score to 0.15, a total of 63 nodes and 136 edges were identified (Fig. 3A). Degree centrality analysis was then performed to identify the top 20 key proteins with the highest degree, which play a central role in the PPI network (Fig. 3B). The network’s average node degree was 4.32, indicating a moderate level of interaction among the proteins. Additionally, the average local clustering coefficient was 0.34, suggesting the presence of some clustering structure in the network, where nodes are relatively more connected to their neighbors.

### 3.4. Candidate drug prediction

**Figure 4.**
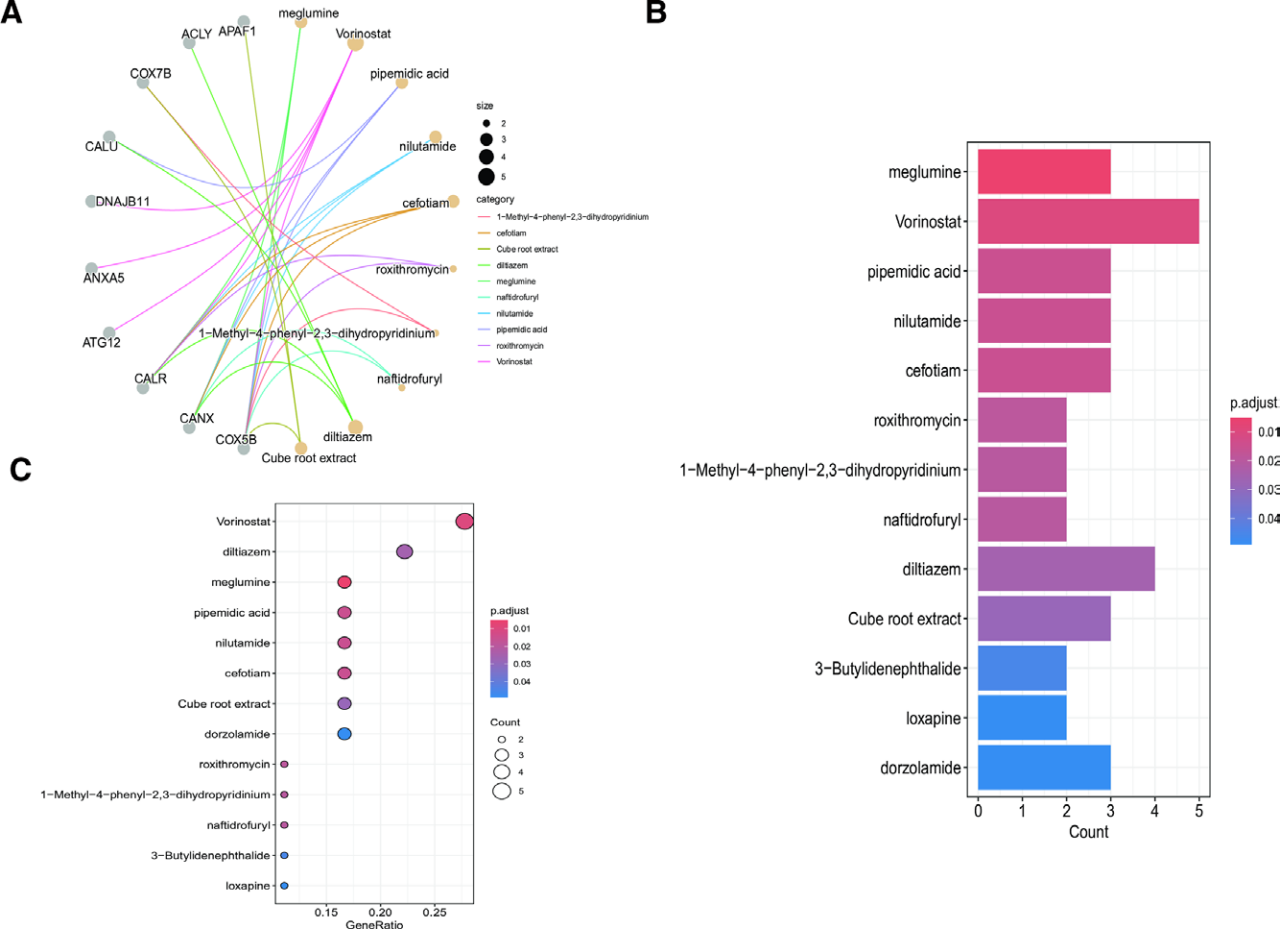
Predicted candidate drugs with significant correlation to SLE, based on DSigDB database; (A) network diagram; (B) bar chart; (C) bubble chart. DSigDB = Drug Signatures Database, SLE = systemic lupus erythematosus.

Using the DSigDB database, we systematically predicted potential therapeutic interventions for SLE. Our analysis identified several compounds showing significant associations (adjusted *P*-value < .05). Among these candidates, meglumine and vorinostat emerged as the most statistically significant, demonstrating robust correlations in our computational screening (Fig. 4A–C). These findings highlight their potential as promising therapeutic agents for SLE intervention.

### 3.5. Molecular docking

**Figure 5.**
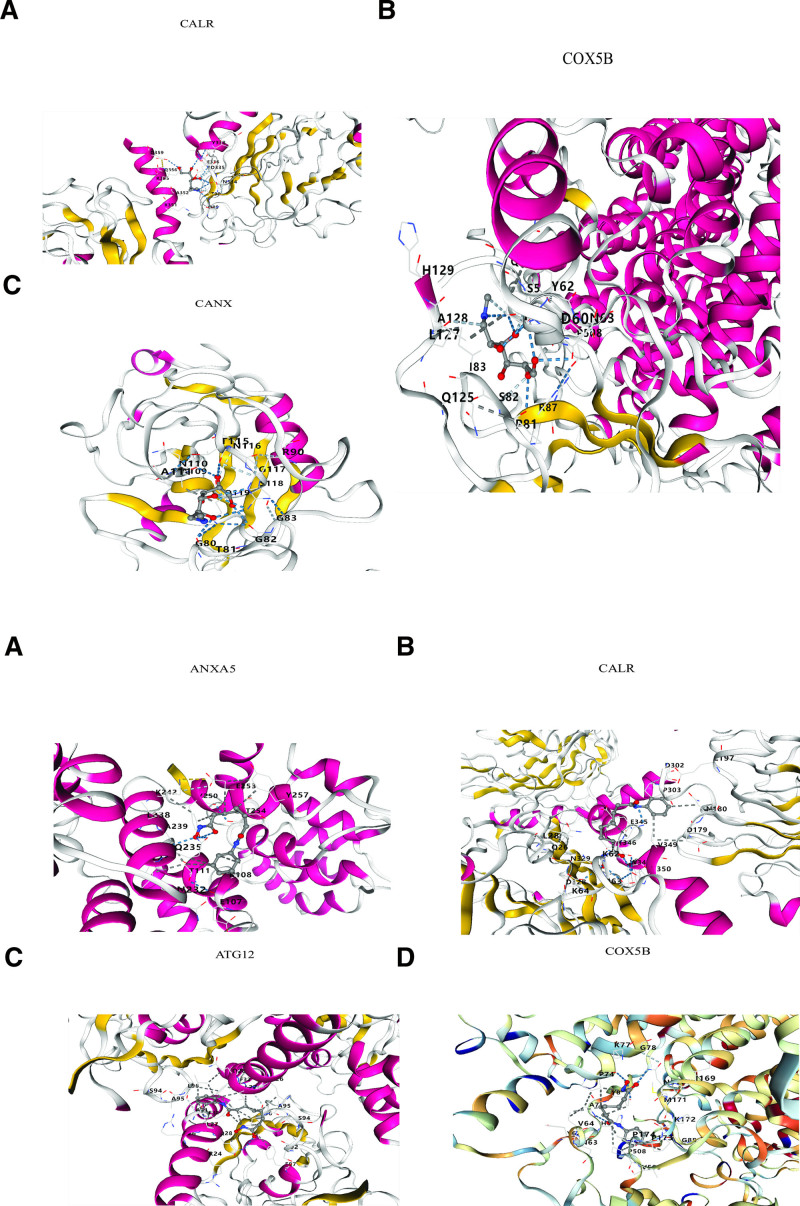
(A) Molecular docking analysis of meglumine interactions with COX5B, CANX, and CALR. (B) Molecular docking analysis of vorinostat interactions with ATG12, COX5B, ANXA5, and CALR. ANXA5 = annexin A5, ATG12 = autophagy-related protein 12, CALR = calreticulin, CANX = calnexin, COX5B = cytochrome C oxidase subunit 5B.

To assess the therapeutic potential of candidate drugs, we performed molecular docking simulations to evaluate their binding affinities with target proteins. Our analysis focused on 2 top compounds showing the strongest statistical correlations. For meglumine, we observed stable interactions with 3 key proteins: cytochrome C oxidase subunit 5B (COX5B), calnexin, and calreticulin (CALR) (Fig. 5A). Vorinostat demonstrated binding potential with 4 targets: autophagy-related protein 12 (ATG12), COX5B, annexin A5 (ANXA5), and CALR (Fig. 5B). Notably, both compounds exhibited favorable binding characteristics, as evidenced by their low binding energies (ranging from −7.5 to −9.2 kcal/mol), indicating the formation of highly stable protein–ligand complexes. These results suggest strong molecular interactions that may underlie their potential therapeutic effects in SLE.

### 3.6. Selection of signature genes

**Figure 6.**
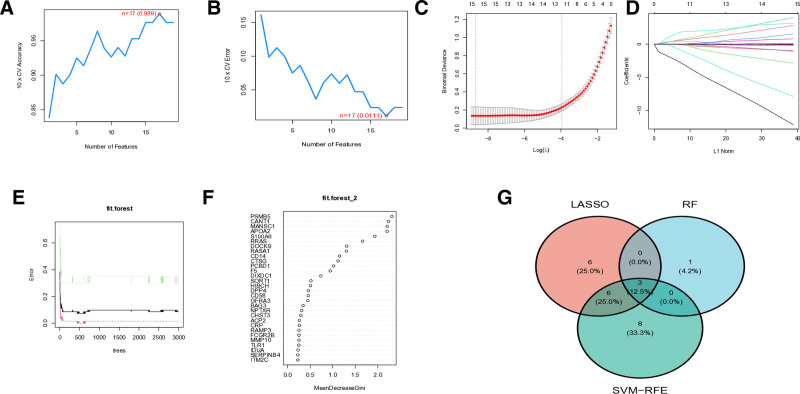
Identification process for signature genes related to SLE. (A and B) Selection and validation of biomarker genes through SVM-RFE method. (C) Feature selection using LASSO regression model. (D) Feature selection optimization under minimum absolute shrinkage conditions. (E) Relationship between the number of decision trees in Random Forest model and error rate. (F) Gene features associated with MDG score. (G) Feature gene screening based on Venn diagram. LASSO = Least Absolute Shrinkage and Selection Operator, MDG = Mean Decrease Gini, SLE = systemic lupus erythematosus, SVM-RFE = support vector machine recursive feature elimination.

Our multi-step feature selection approach systematically identified robust genetic biomarkers for SLE. Initial screening using SVM-RFE yielded 17 significant candidate features (Fig. 6A and B). Subsequent logistic regression refinement narrowed this set to 15 highly SLE-associated genes (Fig. 6C and D). We further validated these findings through Random Forest analysis with feature importance scoring, identifying 4 genes with particularly strong predictive value (Mean Decrease Gini > 2.0; Fig. 6E and F). Comparative analysis of these selection methods via Venn diagram revealed consistent overlap in identified features (Fig. 6G). Through this rigorous multi-algorithm approach, we ultimately identified apolipoprotein A2 (APOA2), MANSC domain-containing protein 1 (MANSC1), and PSMB5 as the most representative and robust feature genes for SLE.

### 3.7. Construction of a SLE diagnostic model based on feature genes

**Figure 7.**
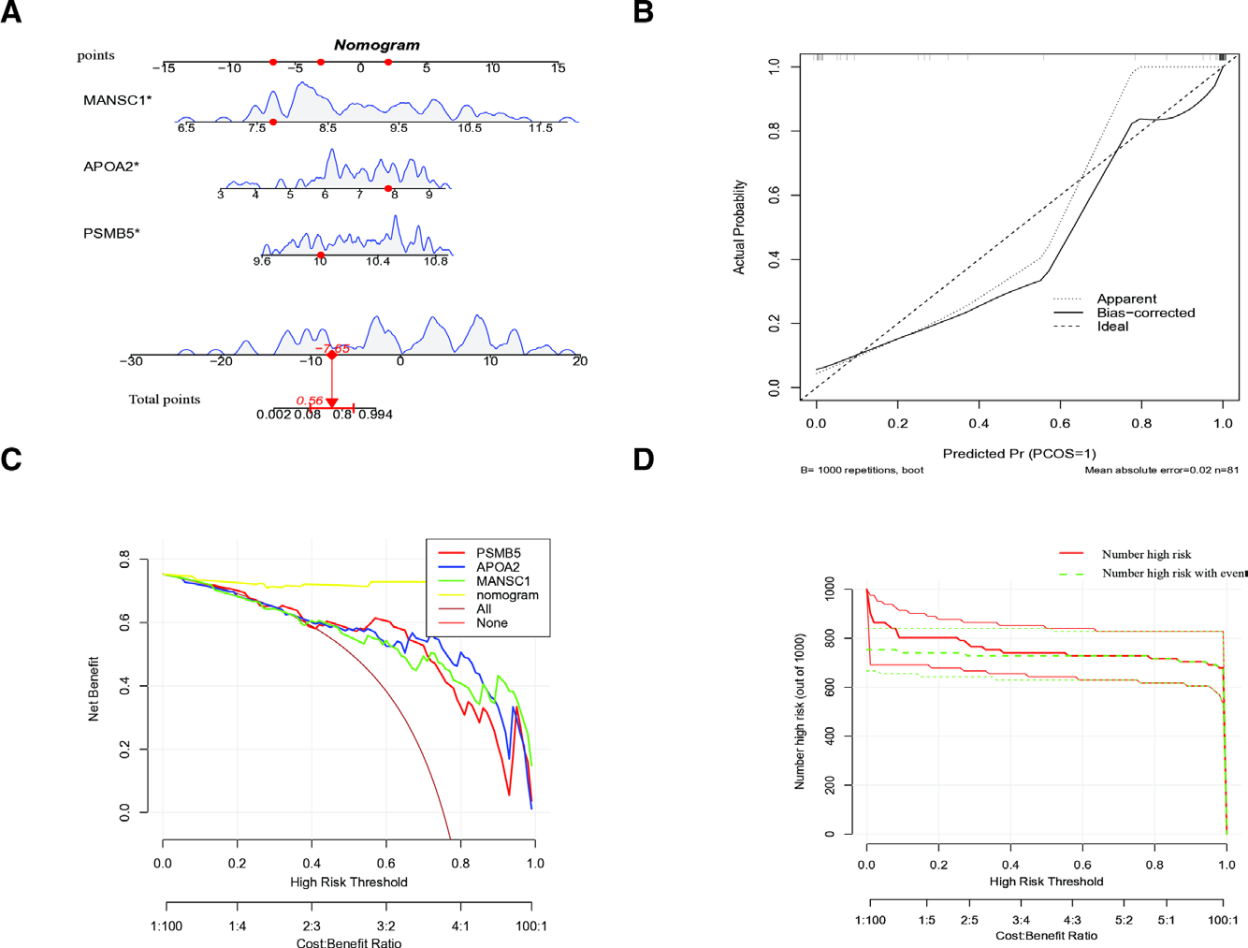
Construction and validation of the diagnostic model for SLE. (A) Nomogram for SLE. (B) Calibration curve of the model. (C) Decision curve analysis. (D) Clinical impact curve. SLE = systemic lupus erythematosus.

We developed a nomogram model for diagnosing SLE based on the feature genes APOA2, MANSC1, and PSMB5. To visualize the performance of this model, we employed R software to generate dynamic modality plots (Fig. 7A), calibration curves (Fig. 7B), and decision curve analysis (DCA, Fig. 7C). The calibration curve demonstrates a strong agreement between the model’s predictions and actual clinical outcomes. Notably, the close alignment between the “high-risk count” curve and the “high-risk count per event” curve indicates the model’s robust ability to predict SLE risk. Taken together, these results highlight the excellent predictive accuracy of the nomogram model for SLE (Fig. 7D).

### 3.8. Assess the performance of the nomogram model using both the training and validation datasets

**Figure 8.**
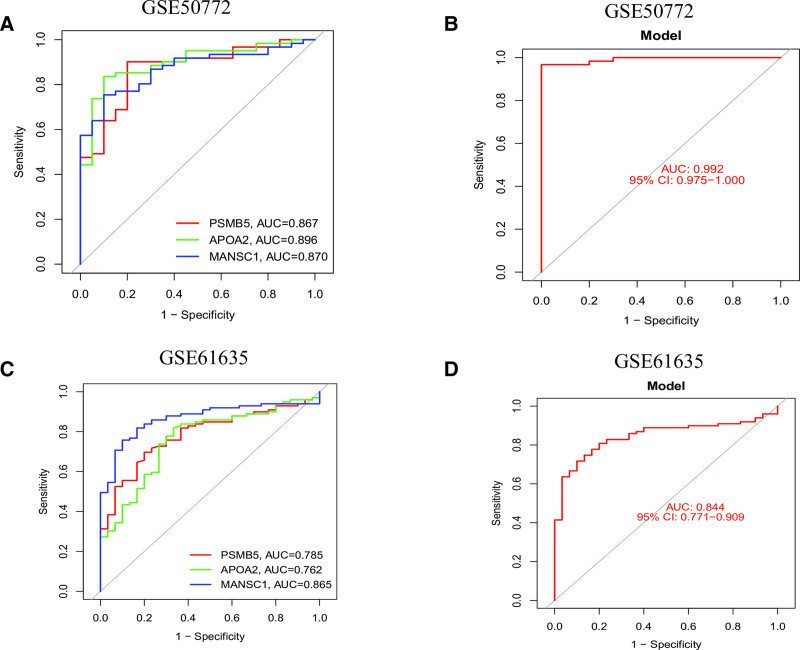
Validation of the nomogram model using ROC curves. (A) ROC and AUC for signature genes in GSE50772. (B) ROC and AUC for nomogram model in GSE50772. (C) ROC and AUC for signature genes in GSE61635. (D) ROC and AUC for nomogram model in GSE61635. AUC = area under the curve, ROC = receiver operating characteristic.

We assessed the diagnostic efficacy of the nomogram model for SLE, constructed based on 3 shared feature genes, by plotting ROC curves with corresponding area under the curve (AUC) values (see Table S1, Supplemental Digital Content, https://links.lww.com/MD/P648). ROC curve analysis in the GSE50772 training cohort revealed AUC values of 0.896, 0.824, and 0.867 for APOA2, MANSC1, and PSMB5, respectively (Fig. 8A). Furthermore, the nomogram model built from these 3 genes demonstrated a high accuracy with an AUC of 0.968 (95% CI: 0.975–1.000) in the training set (Fig. 8B). In the GSE61635 validation cohort, the AUC values for APOA2, MANSC1, and PSMB5 were 0.762, 0.865, and 0.785, respectively (Fig. 8C). The nomogram model also showed strong accuracy in the validation cohort, with an AUC of 0.844 (95% CI: 0.771–0.909) (Fig. 8D). These findings suggest that APOA2, MANSC1, and PSMB5, along with the constructed nomogram model, hold promise as effective biomarkers for the identification and diagnosis of SLE.

### 3.9. Discussion of the specific signaling mechanisms associated with the feature genes APOA2, MANSC1, and PSMB5

**Figure 9.**
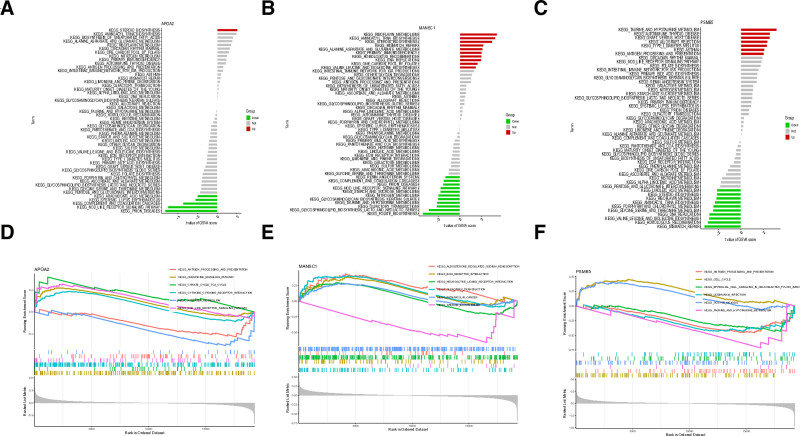
Enrichment analysis of signature genes. (A) GSVA analysis of APOA2; (B) GSVA analysis of MANSC1; (C) GSVA analysis of PSMB5; (D) GSEA analysis of APOA2; (E) GSEA analysis of MANSC1; (F) GSEA analysis of PSMB5. APOA2 = apolipoprotein A2, GSEA = gene set enrichment analysis, GSVA = gene set variation analysis, MANSC1 = MANSC domain-containing protein 1, PSMB5 = proteasome subunit beta 5.

Our investigation into the signaling pathways associated with the 3 central genes (APOA2, MANSC1, and PSMB5) revealed distinct functional implications for each biomarker in SLE pathogenesis. GSVA demonstrated that elevated APOA2 expression showed predominant enrichment in steroid biosynthesis pathways (Fig. 9A), while highly expressed MANSC1 was broadly associated with multiple metabolic and genomic stability pathways, including riboflavin metabolism, branched-chain amino acid biosynthesis, one-carbon metabolism, DNA replication and repair mechanisms, primary immunodeficiency pathways, and tRNA aminoacylation (Fig. 9B). PSMB5 overexpression exhibited particularly strong enrichment in autoimmune-related pathways, including type I diabetes mellitus, transplant rejection mechanisms, and thyroid autoimmunity, along with taurine metabolism (Fig. 9C). Complementary GSEA further delineated these associations, with APOA2 showing significant involvement in inflammatory pathways (chemokine signaling, cytokine–cytokine receptor interactions, and NOD-like receptor signaling) (Fig. 9D), MANSC1 displaying connections to sodium homeostasis and extracellular matrix interactions (Fig. 9E), and PSMB5 demonstrating cell cycle regulatory functions (Fig. 9F). These comprehensive pathway analyses collectively suggest that our identified biomarkers participate in critical pathogenic mechanisms in SLE, influencing immune dysregulation through distinct but interconnected roles in metabolic regulation, genomic maintenance, and autoimmune activation. The pleiotropic nature of these genetic associations underscores their potential as both diagnostic markers and therapeutic targets in SLE management.

### 3.10. Immune infiltration analysis

**Figure 10.**
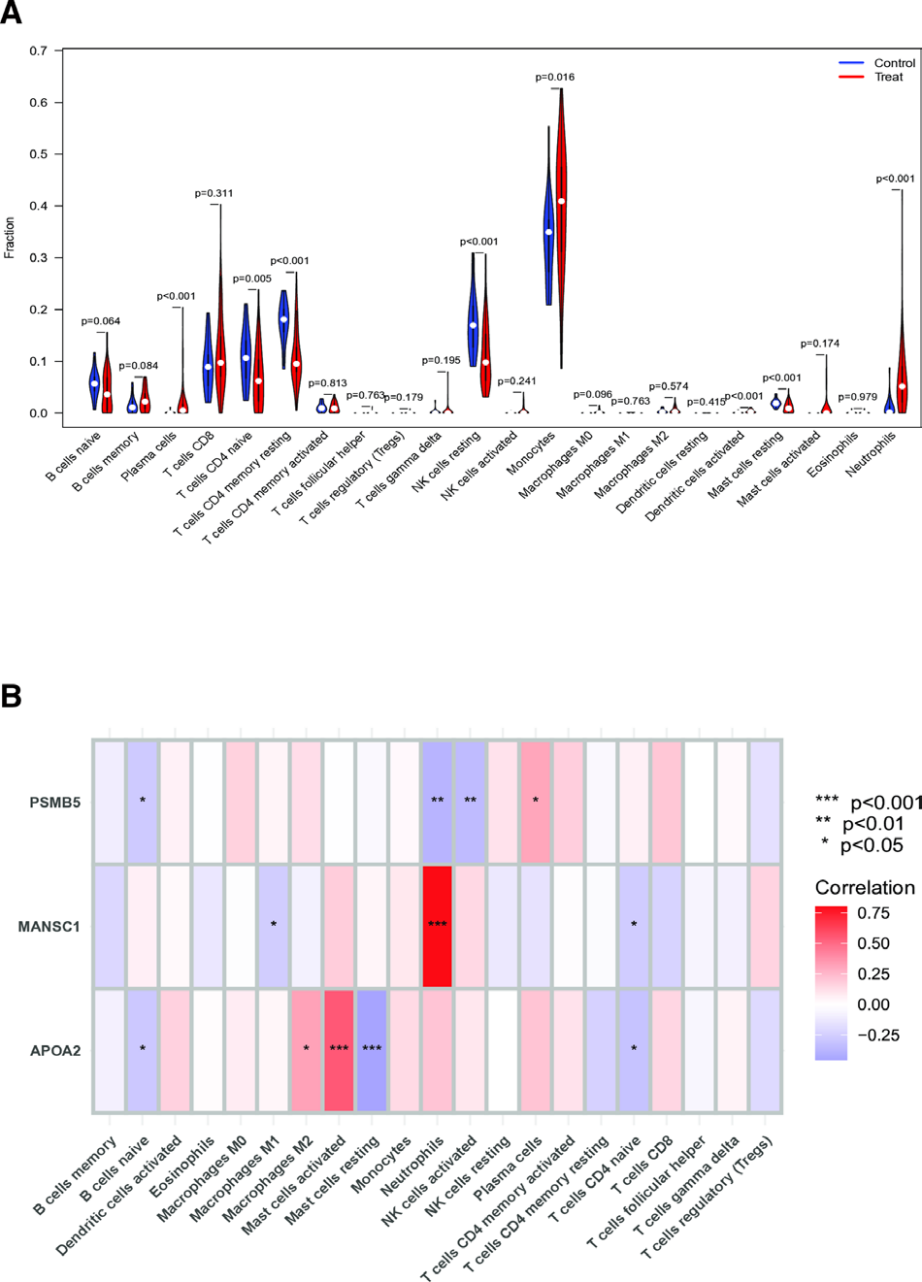
(A) Immune cell infiltration in SLE and healthy controls based on ssGSEA analysis. (B) Correlation between feature genes and immune cell subtypes analyzed by CIBERSORT. SLE = systemic lupus erythematosus, ssGSEA = single-sample gene set enrichment analysis.

Our immune profiling analysis revealed significant differences in immune cell infiltration between SLE patients and healthy controls. Using ssGSEA, we observed substantial enrichment of multiple immune cell populations in SLE, including plasma cells, naive CD4 + T cells, resting memory CD4 + T cells, resting NK cells, monocytes, activated dendritic cells, resting mast cells, and neutrophils (Fig. 10A). These findings suggest that dysregulated immune cell activation contributes substantially to SLE pathogenesis. Subsequent CIBERSORT analysis demonstrated significant correlations between our identified feature genes (APOA2, MANSC1, and PSMB5) and specific immune cell subsets (Fig. 10B). The robust associations revealed by both analytical approaches underscore the central role of inflammatory mechanisms in SLE progression, with our feature genes potentially serving as key regulators of these immune dysregulation processes.

### 3.11. Single-cell visualization of signature gene expression in GSE135779

**Figure 11.**
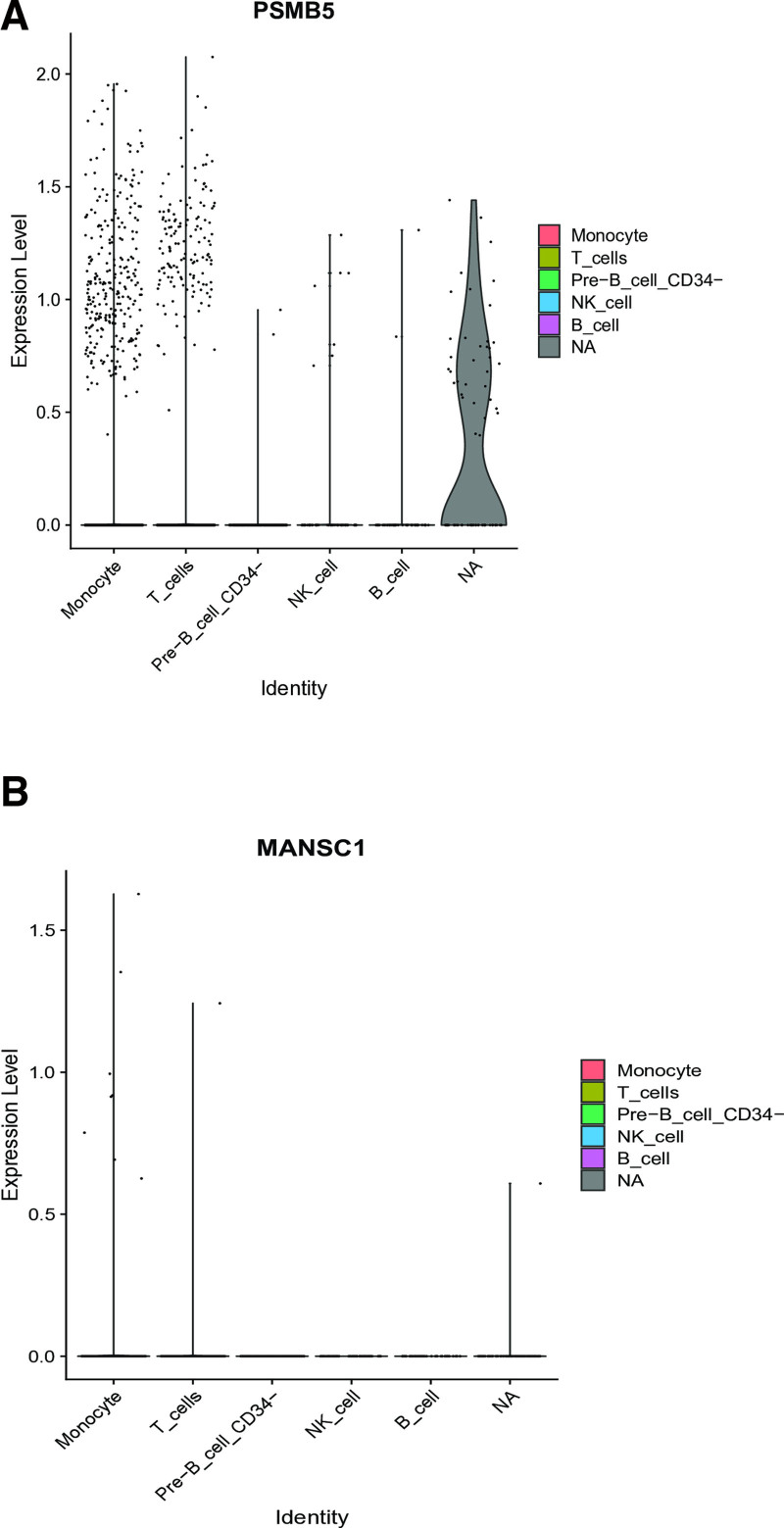
(A and B) Single-cell visualization of MANSC1, PSMB5 expression in GSE135779 dataset. MANSC1 = MANSC domain-containing protein 1, PSMB5 = proteasome subunit beta 5.

Single-cell RNA sequencing analysis of the GSE135779 dataset revealed distinct expression patterns for our candidate biomarkers. PSMB5 and MANSC1 demonstrated significant cell-type specificity, with predominant expression in T cell and monocyte populations (Fig. 11A, B). In contrast, APOA2 expression fell below detectable thresholds in this dataset, precluding detailed cellular localization. These cell-type-specific expression patterns suggest that PSMB5 and MANSC1 may contribute to SLE pathogenesis through specialized roles in adaptive and innate immune cell function, particularly in T cell-mediated immunity and monocyte activity.

## 4. Discussion

SLE is a complex autoimmune disease triggered by interactions among immune cells, cytokines, and genetic factors, characterized by excessive immune cell activation and loss of immune tolerance.^[45]^ Although traditional treatments are effective, they often come with significant side effects and limited efficacy, highlighting the urgent need for new biomarkers and targeted therapeutic strategies.^[46,47]^ This study explores the causal relationship between plasma proteins and SLE using MR analysis, complemented by functional enrichment, immune cell infiltration, and gene screening, providing novel insights for early diagnosis and personalized treatment of SLE.

Our MR analysis revealed that several plasma proteins may play significant roles in the onset and progression of SLE. By leveraging genetic variants, MR analysis assesses causal relationships while controlling for confounding factors, ensuring the reliability of the results. We identified plasma proteins whose genetic variants are significantly associated with SLE risk, particularly those involved in immune response and inflammation regulation. Functional enrichment analysis showed that SLE-associated genes are enriched in biological processes such as glycosaminoglycan metabolism and sulfur compound biosynthesis, both of which are crucial for immune regulation, tissue repair, and damage. KEGG pathway analysis further highlighted the involvement of these genes in lysosomal and glycosaminoglycan biosynthesis pathways, both vital for immune system function, extracellular matrix remodeling, and cellular damage repair. These findings suggest that changes in plasma protein levels may influence the occurrence and immune response of SLE by modulating these key pathways. Among the compounds screened, meglumine and vorinostat showed the most significant statistical associations with target proteins, suggesting their potential as therapeutic agents for SLE. Both drugs interact with proteins involved in key cellular processes such as immune response, inflammation, and cellular stress. Meglumine, commonly used as a contrast agent in imaging,^[48]^ has been observed to possess anti-inflammatory properties. Vorinostat, an HDAC inhibitor, alters gene expression and has been used in the treatment of various cancers and autoimmune diseases.^[49,50]^ Given their roles in modulating immune and inflammatory pathways, we selected these 2 compounds for further analysis to explore their therapeutic potential in SLE. Molecular docking analysis revealed that meglumine and vorinostat form stable complexes with their target proteins, exhibiting low binding energies. Binding energy is a key factor in determining the stability of protein–ligand interactions, and lower binding energies indicate more stable interactions, further supporting the formation of stable complexes. Specifically, meglumine demonstrated significantly lower binding energies with COX5B, calnexin, and CALR, suggesting a strong affinity for these proteins and a potential mechanism for modulating inflammation related to mitochondrial function. Vorinostat interacted with ATG12, COX5B, ANXA5, and CALR: proteins involved in apoptosis regulation, immune response, and calcium signaling, processes critical to SLE pathogenesis. The binding of vorinostat to ATG12 suggests that the drug may influence cellular stress responses, altering immune cell activation. Additionally, the interaction of vorinostat with ANXA5, a protein that regulates inflammation and apoptosis, further supports its potential to modulate immune cell death and survival, crucial in autoimmune diseases.

A unique aspect of this study is the use of scRNA-seq to analyze the expression patterns of key genes (APOA2, MANSC1, and PSMB5) in different immune cell populations in SLE. This analysis provides valuable insights into the immune cell origins of plasma protein expression patterns observed in SLE. We found that PSMB5 and MANSC1 are predominantly expressed in T cells and monocytes, which play central roles in SLE’s immune response and inflammation. These findings suggest that these proteins may regulate immune system function, particularly in the activation and expansion of T cells and monocytes, both critical events in SLE pathogenesis.^[51,52]^ To further investigate SLE’s immune characteristics, we performed immune cell infiltration analysis using ssGSEA. The results revealed significant over-infiltration of multiple immune cell subtypes in the immune systems of SLE patients, particularly plasma cells, naïve CD4 + T cells, resting memory CD4 + T cells, resting NK cells, monocytes, activated dendritic cells, resting mast cells, and neutrophils.^[53]^ These findings suggest that these immune cells play crucial roles in the pathogenesis and immune pathology of SLE, potentially driving immune activation and tissue damage.^[54]^ Correlation analysis between immune cell infiltration and key gene expression revealed that APOA2, MANSC1, and PSMB5 expression levels are strongly associated with the infiltration of specific immune cell subpopulations.^[55]^ These findings suggest that these genes may influence immune dysregulation in SLE by modulating the function and activity of immune cells. Further analysis using GSVA and GSEA explored the specific roles of APOA2, MANSC1, and PSMB5 in SLE immune responses. GSVA analysis indicated that high expression of APOA2 is mainly enriched in steroid biosynthesis pathways, suggesting that APOA2 may impact immune tolerance and balance by regulating steroid hormone synthesis. MANSC1 is linked to various metabolic pathways, including nucleotide metabolism, DNA replication, and immune response, all essential for immune cell proliferation, differentiation, and stress responses. Additionally, high PSMB5 expression is associated with immune-related pathways in SLE, such as the cell cycle and endogenous immune responses, indicating that PSMB5 may influence immune cell proliferation and apoptosis, thereby affecting SLE pathogenesis. GSEA further confirmed the central roles of these genes in SLE immunopathology. High expression of APOA2 is associated with chemokine signaling, cytokine–cytokine receptor interactions, and NOD-like receptor signaling pathways, all critical for immune activation and inflammation. MANSC1 is primarily enriched in pathways related to sodium reabsorption and extracellular matrix receptor interactions, crucial for immune cell migration and inflammatory responses. High expression of PSMB5 is predominantly associated with cell cycle and meiotic division pathways, both critical for immune cell proliferation and differentiation. Another key objective of this study was to develop a diagnostic model based on the feature genes identified in our analysis. Using various machine learning techniques, including SVM-RFE, random forests, and Least Absolute Shrinkage and Selection Operator regression, we identified a set of genes closely associated with SLE risk. APOA2, MANSC1, and PSMB5 were identified as the most important genes, highlighting their potential roles in immune regulation and inflammation. These genes were consistently identified across multiple feature selection methods, confirming their diagnostic potential. The robustness of the model was validated using dynamic modality plots, calibration curves, and ROC curves, demonstrating high predictive accuracy in both training and validation cohorts. These results further support the potential of this model in SLE diagnosis.

### 4.1. Innovation and limitations of the study

The innovation of this study lies in its comprehensive exploration of the causal relationships between plasma proteins and SLE, and their specific roles in immune responses, by integrating MR analysis, GSVA and GSEA, immune cell infiltration, and single-cell data. We not only identified key genes closely associated with SLE but also provided new directions for SLE treatment by predicting potential therapeutic candidates. However, the limitations of this study should be acknowledged. Transcriptomic data has its limitations.^[56,57]^ Although we obtained systematic results through multiple analytical approaches, further experimental data and clinical validation are needed to confirm the actual roles of these genes and targets in SLE. Future studies should validate these findings in patient-derived xenograft models.^[58–60]^ Additionally, while single-cell analysis revealed gene expression profiles in different immune cell types, the precise immune functions of these genes still require further experimental studies for confirmation. Future studies for drug resistance target discovery, such as CRISPR screening,^[61]^ should also be considered.

## 5. Conclusions

APOA2, MANSC1, and PSMB5 are key genes associated with SLE, and the diagnostic model based on these genes shows high predictive accuracy. Single-cell RNA-seq further elucidates their role in immune cells. Our findings suggest that meglumine and vorinostat could be potential therapeutic drugs for SLE, offering new insights into both diagnosis and treatment of the disease.

## Acknowledgments

We extend our heartfelt gratitude for the wealth of resources provided by the public database, which has offered invaluable support to our work and research.

## Author contributions

**Data curation:** Luofei Huang, Han Li.

**Formal analysis:** Luofei Huang.

**Funding acquisition:** Quanzhi Lin.

**Investigation:** Luofei Huang, Quanzhi Lin.

**Methodolgoy:** Jian Shi.

**Project administration:** Han Li, Quanzhi Lin.

**Resources:** Han Li.

**Software:** Han Li, Quanzhi Lin.

**Writing – original draft:** Luofei Huang, Han Li.

**Writing – review & editing:** Jian Shi, Quanzhi Lin.

## Supplementary Material


